# Chemoprofiling as Breeding Tool for Pharmaceutical Use of *Salix*

**DOI:** 10.3389/fpls.2021.579820

**Published:** 2021-04-01

**Authors:** Nadja Förster, Kyriaki Antoniadou, Matthias Zander, Sebastian Baur, Verena Karolin Mittermeier-Kleßinger, Corinna Dawid, Christian Ulrichs, Inga Mewis

**Affiliations:** ^1^Division Urban Plant Ecophysiology, Humboldt-Universität zu Berlin, Berlin, Germany; ^2^Chair of Food Chemistry and Molecular Sensory Science, Technical University of Munich, Freising, Germany; ^3^Bavarian Center for Biomolecular Mass Spectrometry, Technical University of Munich, Freising, Germany

**Keywords:** chemoprofiling, clustering, phenolics, salicylates, *Salix* crosses, willow

## Abstract

Willow bark is traditionally used for pharmaceutical purposes. Evaluation is so far based on the salicylate content, however, health promoting effects of extracts might be attributed to the interaction of those salicylates with other compounds, which support and complement their action. So far, only *S. purpurea*, *S. daphnoides*, and *S. fragilis* are included in pharmaceutical extracts. Crossing with other species could result in a more diverse secondary metabolite profile with higher pharmacological value. With the help of targeted inter- and intraspecific crossing, new chemotypes were generated, whereby nine different *Salix* genotypes (*S. alba*, *S. daphnoides*, *S. humboldtiana*, *S. lasiandra*, *S. nigra*, *S. pentandra*, *S. purpurea*, *S. x rubens*, *S. viminalis*) were included in the study. Based on substances known for their health promoting potential and characteristic for *Salix* (selected phenolic compounds including salicylates), a targeted metabolomics analysis and clustering of 92 generated *Salix* clones was performed revealing four different cluster/chemoprofiles. In more specific, one group is formed by *S. daphnoides* clones and inter- and intraspecific hybrids, a second group by *S. viminalis* clones and inter- and intraspecific hybrids, a third group generally formed by *S. alba*, *S. pentandra*, *S. x rubens*, and *S. lasiandra* clones and hybrids, and a fourth group by *S. purpurea* clones and inter- and intraspecific hybrids. Clustering on the basis of the selected phenolic compounds can be used for identifying *Salix* clones with a different compound profile. New combinations of secondary plant metabolites offer the chance to identify *Salix* crosses with improved effects on human health.

## Introduction

Bark of *Salix* spp. is used for pharmaceutical purposes to treat headaches as well as fever, rheumatic diseases, arthritis, and relieve low back pain. These anti-inflammatory and anti-oxidative effects were attributed mostly to specific bark compounds, the salicylates, which have been identified in selected *Salix* species (Meier et al., 1987; Kammerer et al., 2005). Therefore, herbal remedies were obtained from *Salix* species, which contain minimum 1.5% total salicylic derivatives as, for example, postulated for *Salix purpurea*, *Salix daphnoides*, and *Salix fragilis* (ESCOP, 2017). To treat low back pain and mild rheumatic conditions, ESCOP recommends a daily oral dose of up to 240 mg equivalent salicin/day (salicylates hydrolyzed to salicin). Using Salicis cortex for treating arthritis and rheumatism is widespread accepted and analyzed intensively as reviewed in Sulima and Przyborowski (2019). For example, according to an open, multicentric observational study with reference treatment where patients were treated with a standardized willow bark extract, results show that using the willow bark extract was comparable in effectiveness to standard therapies for treating mild or fairly severe cases of gonarthrosis and coxarthrosis (Beer and Wegener, 2008).

Additionally to the salicylates, identified as biological markers for activity, other groups of secondary plant metabolites are known in the bark of willows, like e.g., flavonoids, flavan-3-ols, and oligomeric procyanidins (Shao, 1991; Jürgenliemk et al., 2007; Förster et al., 2008). *Salix* species can differ widely in their secondary plant metabolite profiles. Additionally, current studies have clearly shown that bioactive effects of bark extracts cannot be exclusively traced back to the salicylates. Other bark compounds can support and complement their action. Further, other identified compounds are also of relevance (Schmid et al., 2001; Khayyal et al., 2003, 2005; Lardos et al., 2004; Nahrstedt et al., 2007; Ishikado et al., 2013; Enayat and Banerjee, 2014). Khayyal et al. (2003), for instance, analyzed the effect of willow bark extract and different fractions in comparison to acetylsalicylic acid (ASS) on edema in the rat paw. The extract and ASS inhibit the edema in the same degree. Analysis of the single fractions show that additionally to the salicin containing fractions, fractions containing polyphenols were active and show anti-inflammatory effects. Nahrstedt et al. (2007) analyzed five different extract fractions (A–E; A: mainly unidentified polyphenols, B: unidentified polyphenols and flavonoids, C: flavonoids, D: salicin, and E: proanthocyanidins) in *in vitro* and *in vivo* studies and identified efficacies of willow bark extract fractions are mediated by polyphenols, like proanthocyanidins and flavonoids, as well. In *in vitro* studies of Ishikado et al. (2013) it was found that the activation of the ARE promoter, proven for willow bark extracts, was not mediated by salicin or its metabolites. Salicin free extracts showed an activation and thus an induction of antioxidative enzymes and prevention from oxidative stress via activation of Nrf2. Additionally, in comparison to single compounds, synergetic effects of different compounds in the bark are conceivable (Nahrstedt et al., 2007; Enayat et al., 2013) and well-known for drugs produced from natural products (Liu, 2003; Ulrich-Merzenich et al., 2010). Durak and Gawlik-Dziki (2014) described synergistic effects of active compounds in coffee and willow, known as valuable sources for chlorogenic acid and salicin derivates, respectively. Due to the point that both substances are known to be present in high contents in specific *Salix* species, the presumption of synergism is strengthened. Also, synergistic effects of flavonoids and phenolic acids were determined (Hajimehdipoor et al., 2014), both compound classes are known for *Salix*.

Considering the background of an aging population along with increasing numbers of age-related diseases like rheumatism, arthritis, etc., targeted *Salix* crosses, which show new combinations of diverse secondary plant metabolites, can be used to produce bioactive willow bark extracts with an improved medicinal potential. According to the European Union herbal monograph on *Salix* (EMA/HMPC/80630/2016) the species *S. purpurea*, *S. daphnoides*, and *S. fragilis* are allowed to be included in pharmaceutical extracts. Other species might be of interest for usage in the future. For example, in the bark of *Salix pentandra* high contents of 2‘-*O*-Acetylsalicortin, an acetylated form of salicortin, is present (Förster, 2010). Analysis of acetylated and non-acetylated glucosinolates of *Moringa oleifera* showed that the acetyl group can have a decisive role promoting biological activity (Förster et al., 2016). Piatczak et al. (2020) examined a strong anti-oxidative activity of *Salix alba* bark extracts, a willow species with no or less contents of salicylic acids. Additionally to their differing bark compound profile, shrub willow is an energy crop and is known to be fast-growing and high-yielding. Many studies have highlighted the complex, multigenic basis for heterosis in inbred crops (Carlson, 2018). Hybridization of *Salix* is used to generate crosses with higher biomass yield and growth. A targeted creation of *Salix* crosses with a diverse compound profile was not in focus of work until now.

The aim of the study was to perform targeted crossing of *Salix* species to generate different chemotypes. Next to two species named in the monograph (*S. purpurea*, *S. daphnoides*), seven more *Salix* genotypes were included: *Salix alba*, *Salix humboldtiana*, *Salix lasiandra*, *Salix nigra*, *Salix pentandra*, *Salix x rubens*, *Salix viminalis. Salix* species were chosen based on a diverse bark chemical profile as well as the possibility to generate vital crosses. With the help of targeted inter- and intraspecific crossing, 64 new *Salix* crosses were generated. Based on known bioactive compound groups in the *Salix* bark as well as single characteristic metabolites in the selected willow species, all *Salix* species and crosses were divided into different clusters. This targeted approach was performed to confirm bioactivity according to pharmaceutical standards. Next to salicylates, there exists a general knowledge about possible health promoting effects of phenolic compounds also known for *Salix* bark as reviewed for example for flavonoids (Panche et al., 2016), proanthocyanidins (Cires et al., 2017), and phenolic acids such as chlorogenic acid and caffeic acid (Sato et al., 2011; Naveed et al., 2018). The presence of dissimilar groups of flavonoids may change the type of a pharmacological activity in the bark, from the increase of antioxidant effect (Evans, 2000). For isosalipurposide, in an *in vivo* assay of two-stage carcinogenesis on mouse skin a delayed formation of papillomas was found (Yagura et al., 2008). Additionally, the authors postulated anti-proliferative and anti-carcinogenic activity. Chalcones (isosalipurposid and its derivatives) and flavanones (naringenin and its glucosides) are known to enhance spasmolytic and anti-inflammatory activity (Bandgar et al., 2012; Letafat et al., 2013). For catechin and epicatechin a lot of pharmacological effects like anti-oxidative, anti-carcinogenic, or cardioprotective effects were reported (Ganeshpurkar and Saluja, 2020). Due to the point of characteristic/specific compounds for *Salix* (in general and in single species) as well as known health promoting potential of single ingredients as well, the following secondary plant compounds were included in the targeted approach: flavan-3-ols (catechin and epicatechin), flavonoids (eriodictyol-7-*O*-glucoside, two isomers of naringenin-5-*O*-glucoside, naringenin-7-*O*-glucoside, luteolin-7-*O*-glucoside, quercetin-hexoside, isosalipurposide, and ampelopsin), salicylates (salicin, salicortin, 2‘-*O*-acetylsalicortin, 2‘-*O*-acetylsalicin, and tremulacin), other phenolic compounds (triandrin, two caffeic acid derivatives, two purpurein isomers, salireposide, and syrengin). Goal of the study was to identify new *Salix* crosses with diverse compound profiles, which could provide the basis for willow extracts with an improved value on human health. These (optimized) extracts could be used as alternative to non-steroidal anti-inflammatory drugs (NSAIDs) for treating joint and spine related inflammatory pain—as herbal remedies are often better tolerated and have less side effects (Maroon et al., 2010).

## Materials and Methods

### Chemicals

The following compounds used for preparing extracts by targeted analysis of phenolic compounds and eluents were obtained commercially: resorcinol (Fluka, Germany), methanol (HPLC grade, VWR BDH Chemicals, Poole, United Kingdom), tetrahydrofuran (HPLC grade, VWR BDH Chemicals, Poole, United Kingdom), formic acid (Merck KGaA, Darmstadt, Germany), and orthophosphoric acid (85%, VWR BDH Chemicals, Poole, United Kingdom). Water used for extract and eluent preparation was purified by a Milli-Q Integral 10 system (Millipore, MA, United States).

### Collection of *Salix* Species

Hardwood cuttings from the following *Salix* species originated in Germany, Poland, Austria, Sweden, Romania, Hungary, United Kingdom, and the United States were collected between 2006 and 2009: *Salix alba*, *Salix daphnoides*, *Salix humboldtiana*, *Salix lasiandra*, *Salix nigra*, *Salix pentandra*, *Salix purpurea*, *Salix x rubens*, *Salix viminalis* (Supplementary Table). Clones were established and cultivated at Humboldt-Universität zu Berlin (exceptions see Supplementary Table). Parental forms were planted in 2012 and crosses in 2015 (for VI3_h, VI6, HU1, and SN1 not known). To include crossable species with a diverse compound profile in the crossing experiment, in pre-tests the profile of selected phenolics in the *Salix* bark (see targeted analysis) was analyzed (Shao, 1991; Förster, 2010; Köhler, 2016; Bubner et al., 2018).

### Inter- and Intraspecific *Salix* Crosses

Crosses were generated from matured stock collections from the different *Salix* species whereby branches of males were cut in spring. After flowering, the pollen was collected and stored at 4°C. Catkins of females were bagged before flowering and directly pollinated on the plant or were pollinated in crossing chambers with a sterile brush. The ripened capsules are used as seeds for further cultivation.

### Propagation and Cultivation of *Salix* Species and Crosses

Collected cuttings from *Salix* species were inserted and further cultivated on open field areas in Berlin or Zepernick, Germany (6–9 cuttings per species). Seeds from new generated *Salix* crosses were sown in pots and cultivated in the greenhouse for a few weeks until they were transferred to the open field areas in Berlin or Zepernick. too. Only viable seedlings with a vigorous growth were selected for planting in the open field areas. From each seedling hardwood cuttings were generated to have 6–9 cloned single plants for each genotype. Based on the vitality of the seedlings from the different crossing combinations 1–12 descendants were planted. The planting pattern was 300 cm between and 50 cm within the rows. Between different clones a distance of 150 cm was used. Besides an annual pruning back to the trunk, plants were watered at severe drought and mulched to reduce weed pressure.

To analyze inter- as well intraspecific differences in the compound profile of *Salix* bark, 92 clones mostly cultivated in Berlin or Zepernick, Germany (parental and filial generation) were included in the study (Supplementary Table). Bark material of willow species which were not yet in focus of our work was harvested in collections of the Thünen-Institute in Waldsieversdorf, Germany, Wriezen, Germany, or in Garzau, Germany (Supplementary Table).

For the chemical analysis of bark material 1-year-old branches of the *Salix* plants were cut off in August 2016. Due to the point that clone variability was not in focus of the present work, from each genotype (species and crosses) a composite sample from bark material of 3–5 plants generated from the hardwood cuttings from each mother plant was used. Bark from the branches was peeled at a height from 10 to 100 cm, frozen (−80°C), and immediately lyophilized.

### Targeted Analysis to Determine Phenolic Compounds in the *Salix* Bark

For the extraction of phenolics a method described by Förster et al. (2008) was used. Briefly, 50 mg pulverized bark material was extracted with 750 μL 80% methanol and 100 μl resorcinol (50 mM, internal standard) in an ultrasonic bath with ice water for 10 min. After centrifugation (6,000 rpm, 5 min, 4°C) the supernatant was collected and the pellet was re-extracted with 500 μL of the extraction solution twice. The combined supernatants were concentrated in a vacuum concentrator to near dryness and refilled with ultrapure water up to 1 mL. The extract was filtered using SpinX tubes (0.22 μm) and stored at −20°C until HPLC analysis. The HPLC system consisted of a DIONEX P680 pump, an ASI-100 auto sampler, a TCC-100 thermally regulated column department and an UltiMate 3000 Photodiode Array Detector. The software Chromeleon 6.8 was used for peak evaluation. Reversed phase chromatography was carried out on an Acclaim PolarAdvantage C16 column (3 μm, 120 Å, 2.1 × 150 mm, Thermo Fisher Scientific) protected by a pre-column (5 μm, 120 Å, 2 × 10 mm, Thermo Fisher Scientific). Eluents used for HPLC analysis were (A) 2% tetrahydrofuran, 0.5% phosphoric acid in ultrapure water and (B) 100% methanol. The extracts were analyzed with a flow rate of 0.35 mL/min and the following gradient program: 0% B (0–5 min), 0–15% B (5–10 min), 15–25% B (10–20 min), 25–35% B (20–30 min), 35–50% B (30–40 min), 100% B (40–42 min), 100–0% B (42–44 min), and 0% (44–49 min) injecting each sample two times (technical replicates). Injection volume was 10 μL and peak detection was carried out at 270 nm. Phenolics were quantified against internal standard resorcinol (50 mM, Fluka). Commercially available or isolated standards of single compounds were used as reference. Response factors (RF) in relation to the internal standard resorcinol were used to correct for absorbance difference (RF salicin: 1.73, RF salicortin: 0.34, RF 2′-*O*-acetylsalicortin: 2.98, RF tremulacin: 4.53, RF eriodictyol-7-*O*-glucoside: 0.16, RF naringenin-7-*O*-glucoside: 0.27, RF luteolin-7-*O*-glucoside: 0.07, RF ampelopsin: 0.22, RF catechin: 0.34, RF epicatechin: 0.86, RF syrengin: 0.23). If a standard was not available, RF of a compound with a similar chemical structure was used (2′-*O*-acetylsalicin: RF as 2′-*O*-acetylsalicortin 2.98, naringenin-5-*O*-glucoside I + II: RF as naringenin-7-*O*-glucoside 0.27, quercetin-hexoside: RF as isoquercitrin 0.08, triandrin and purpurein I + II: RF as coumaric acid 0.17, caffeic acid derivatives: RF as caffeic acid 0.27) or set as 1 (isosalipurposide, salireposide). Qualitative analysis and identification of phenolics was based on their retention times, specific UV-spectra (Shao, 1991) and mass spectrometry (characteristic mass fragment ions by HPLC-DAD-ESI-MS^3^, [M-H]^–^). The phenolic content was calculated in mg/g dry weight (DW).

### Cluster Analysis of *Salix* Analyzed by the Targeted Approach

Grouping analysis of quantitative compound data was done by SPSS Statistics Version 24 using the Ward’s method with Euclidean distance as a hierarchical clustering method. Based on the formed dendrogram, four clusters were used to describe the dataset best.

## Results

To determine the content of the selected phenolic compounds in the *Salix* bark, a targeted metabolomic analysis was performed. The following phenolic compounds were identified and quantified: five salicylates (salicin, salicortin, 2‘-*O*-acetylsalicortin, 2′-*O*-acetylsalicin, and tremulacin), eight flavonoids (eriodictyol-7-*O*-glucoside, two isomers of naringenin-5-*O*-glucoside, naringenin-7-*O*-glucoside, luteolin-7-*O*-glucoside, quercetin-hexoside, isosalipurposide, and ampelopsin), two flavan-3-ols (catechin and epicatechin), and seven other phenolic compounds (triandrin, two caffeic acid derivatives, two purpurein isomers, salireposide, and syrengin). The different *Salix* species and crosses differ in their profile of detected phenolic compounds (Tables 1–4). Total phenolic contents vary between 1.70 (VI6, Table 2) and 42.31 mg/g DW (DA2, Table 1). *S. daphnoides*, *S. purpurea*, and *S. pentandra* clones show generally highest phenolic contents (f. e. DA5 17.77 mg/g DW, PU2 26.30 mg/g DW, Table 1; PE1 38.79 mg/g DW, Table 2), and *S. alba* and *S. viminalis* lowest (VI4 3.97 mg/g DW, Table 2; AL5 2.59 mg/g DW, Table 3). Whereas, *S. daphnoides* clones showed high contents of salicylates, especially salicortin (e.g., DA2 with 11.77 mg/g DW), no salicylates could be found in *S. viminalis* (except VI1). *S. pentrandra* clones contained high contents of 2′-*O*-acetylsalicortin (e.g., PE1 with 33.22 mg/g DW). *S. alba*, *S. lasiandra*, and *S. humboltiana* showed just low salicylate contents (salicin, 2′-*O*-acetylsalicortin). While, *S. viminalis* was characterized by high contents of triandrin (e.g., VI3_h with 10.37 mg/g DW), *S. purpurea* contained high levels of total flavonoids and other phenolic compounds like purpurein and salireposide.

**TABLE 1:** Phenolic compound profile of clones of *S. viminalis*, *S. daphnoides*, and their crosses.

	Phenolic compounds (mg/g DW)																				

	Salicylates			Flavonoids								Flavan-3-ols		Other phenolic compounds						Total	Cluster targeted
				
	Salicin	Salicortin	Tremulacin	Eriodictyol-7-glc	Naringenin-5-glc I	Naringenin-5-glc II	Naringenin-7-glc	Luteolin-7-glc	Quercetin-hexoside	Isosalipurposide	Ampelopsin	Catechin	Epicatechin	Triandrin	Caffeic acid der. I	Caffeic acid der. II	Purpurein	Salireposide	Syrengin		
*S. daphnoides* (DA2 × DA3)	2.87	3.37	3.98	0.05	0.81	1.00	0.89	0.06	0.03	1.13		0.87			0.09	0.50	0.07		1.31	17.02	1
*S. viminalis* (VI2)											0.38	0.67		4.74					0.23	6.02	2
**Crosses**																					
1	1.26	3.53	6.86	0.05	0.86	0.71	1.40	0.20	0.03			1.08			0.78	0.40			0.52	17.68	1
2	2.37	8.08	11.87	0.11	1.34	1.59	2.18	0.09	0.03	1.71		1.35	0.66		1.17	0.37			0.70	33.62	1
3	1.98	7.33	8.01	0.33	1.52	2.13	3.30	0.07		2.83		1.73	0.83		0.53	0.56			0.69	31.84	1
4	0.93	2.83	5.15	0.04	0.31	0.48	0.42	0.08	0.04			0.79	0.66		0.90	0.76			0.38	13.77	1
5	1.68	5.10	4.18	0.10	1.18	1.41	1.35	0.17	0.05	1.39		1.27	1.05		0.93	0.78			0.60	21.23	1
*S. daphnoides* (DA2)	2.42	11.77	11.10	1.37	1.86	2.21	2.69	0.10	0.03	5.17		1.26	0.56		0.60	0.72			0.45	42.31	1
*S. daphnoides* (DA3)	0.89	1.51	3.45	0.12	0.90	1.15	1.63	0.04	0.05			1.15	0.54		1.09	1.14			2.12	15.78	1
**Crosses**																					
1	1.45	6.28	9.34	0.15	0.88	1.04	1.72	0.02	0.02	1.96		1.58	0.59		0.52	0.76			0.74	27.05	1
2	1.69	7.14	7.63	0.13	1.16	1.65	1.42	0.04	0.02	2.52		1.39	0.65		0.65	0.68			0.73	27.50	1
3	1.41	5.03	3.30	0.17	1.07	1.43	1.42	0.05		1.69		1.31	0.29		0.35	0.35			0.56	18.43	1
4	1.35	4.08	9.51	0.05	0.48	0.59	0.72	0.06	0.02	0.80		0.91			0.51	0.60			0.66	20.35	1
5	1.85	7.35	8.59	0.15	1.36	1.93	1.41	0.04	0.02	3.03		1.76	0.43		0.55	0.54			0.58	29.59	1
6	1.29	0.55	7.97	0.03	0.65	0.72	1.12	0.03	0.02	1.05		1.11			0.55	0.46			0.66	16.21	1
7	1.63	4.52	7.80	0.07	1.14	1.48	1.10	0.07	0.01	1.49		1.67	0.29		0.28	0.52			0.69	22.76	1
8	1.84	7.75	6.97	0.17	1.86	2.18	2.29	0.07	0.01	3.79		1.90	0.57		0.57	0.66			0.94	31.57	1
*S. daphnoides* (DA5)	2.50	7.39	6.38	0.07	1.51	2.42	0.75	0.03	0.05	2.59		1.44			0.78	0.92			0.94	27.77	1
*S. purpurea* (PU2)	1.01	3.54	8.09	0.45	2.44	2.12	0.46	0.28	0.22	1.37	0.11	3.58					0.07	2.12	0.44	26.30	4
**Crosses**																					
1	2.92	6.95	3.09	0.22	1.77	2.80	0.76	0.02	0.14	2.91		1.56			0.77	0.51	0.10		1.06	25.58	1
2	2.41	4.07	5.82	0.11	0.80	1.14	1.02	0.04	0.11	0.58		1.87			0.88	1.00	0.04		1.47	21.37	1

**glc, glucoside; der., derivate; S., Salix. Standard deviation of all compound contents varied between 0.00 and 0.16.**

**TABLE 2:** Phenolic compound profile of clones of *S. viminalis*, *S. daphnoides*, *S. purpurea*, *S. humboldtiana*, *S. nigra*, *S. pentandra*, and their crosses.

	Phenolic compounds (mg/g DW)																						

	Salicylates					Flavonoids								Flavan-3-ols		Other phenolic compounds						Total	Cluster targeted
				
	Salicin	Salicortin	2′-O-Acetylsalicortin	2′-O-Acetylsalicin	Tremulacin	Eriodictyol-7-glc	Naringenin-5-glc I	Naringenin-5-glc II	Naringenin-7-glc	Luteolin-7-glc	Quercetin-hexoside	Isosalipurposide	Ampelopsin	Catechin	Epicatechin	Triandrin	Caffeic acid der. I	Caffeic acid der. II	Purpurein I	Salireposide	Syrengin		
*S. viminalis* (VI1)	0.47										0.02		0.09	0.89		8.82					4.49	14.78	2
*S. daphnoides* (DA1)	2.24	6.40			11.36	0.03	1.31	1.92	1.39	0.08	0.04	2.36		1.50	1.04		1.05	1.12			1.28	33.12	1
**Crosses**																							
1	1.03	3.18			6.75	0.08	0.75	1.13	1.70	0.11	0.17	1.29		1.99	0.84	0.77	1.12	1.10			3.04	25.05	1
2											0.03		0.32	0.46		4.03					0.29	5.13	2
3	0.04										0.07		0.65	0.70		3.82					0.43	5.71	2
4	0.05										0.05		0.44	0.82		7.14					0.27	8.77	2
5	0.10										0.04		0.52	0.81		3.27					0.57	5.31	2
6	0.07										0.04		0.73	0.92		5.81					0.43	8.00	2
7											0.03		0.18	0.55		3.64					0.25	4.65	2
8	0.24										0.03		0.34	0.53		2.16					0.26	3.56	2
9	0.78	1.37				0.03	0.26	0.26	0.58	0.03	0.06			1.42		2.81		0.08			0.97	8.65	3
*S. purpurea* (PU4)	0.74	1.90			1.84	0.79	1.12	1.32	0.23	0.13	0.03	0.96	0.09	2.32					0.10	1.41	0.56	13.54	4
*S. viminalis* (VI2)													0.38	0.67		4.74					0.23	6.02	2
**Crosses**																							
1	0.38	0.67				0.08	0.50	0.48	0.10	0.01		0.34	0.26	2.02		3.94				1.20	0.12	10.10	3
2	0.27					0.04	0.43	0.54	0.09	0.02	0.02	0.16	0.33	1.11		1.70				0.56	0.28	5.55	3
*S. humboldthiana* (HU1)	0.69					0.01	0.33	0.40			0.09		1.47	1.24		2.98				0.26	0.65	8.12	2
*S. viminalis* (VI6)	0.29										0.03		0.13	0.85		0.01					0.39	1.70	3
Crosses																							
1	0.81					0.01	0.27	0.34			0.02		0.10	1.06		5.19					0.35	8.15	3
2	0.50					0.01	0.52	0.76			0.05		0.20	0.68		3.99					0.35	7.06	3
*S. nigra* (SN1)											0.02		0.25	0.88		3.02					0.29	4.46	2
*S. pentandra* (PE1)	0.16		33.22	4.38							0.03			0.41	0.25						0.34	38.79	3
Crosses																							
1			1.17								0.03		0.21	0.97		8.07					0.23	10.68	2
*S. viminalis* (VI4)											0.03		0.46	0.55		2.86					0.07	3.97	2
*S. viminalis* (*schwerinii × viminalis*) (VI3_h*)													0.46	0.78		10.37					0.36	11.97	2
**Crosses**																							
1													0.33	0.32		6.09					0.13	6.87	2
2											0.06		0.42	0.51		9.42					0.23	10.64	2
*S. viminalis* (VI5)													0.20	0.64		8.36					0.14	9.34	2
*S. viminalis* (VI2)													0.38	0.67		4.74					0.23	6.02	2
**Crosses**																							
1													0.75	0.43		4.03					0.23	5.44	2
2													0.37			5.75					0.17	6.29	2
3													0.34	0.89		6.12					0.19	7.54	2
4													0.37	0.53		4.11					0.15	5.16	2

**glc, glucoside; der., derivate; S., Salix. Standard deviation of all compound contents varied between 0.00 and 0.16.**

**TABLE 3:** Phenolic compound profile of clones of *S. alba*, *S. x rubens, S. purpurea*, *S. humboldtiana*, *S. lasiandra*, *S. pentandra*, and their crosses.

	Phenolic compounds (mg/g DW)																							

	Salicylates					Flavonoids								Flavan-3-ols		Other phenolic compounds							Total	Cluster targeted
				
	Salicin	Salicortin	2′-O-Acetylsalicortin	2′-O-Acetylsalicin	Tremulacin	Eriodictyol-7-glc	Naringenin-5-glc I	Naringenin-5-glc II	Naringenin-7-glc	Luteolin-7-glc	Quercetin-hexoside	Isosalipurposide	Ampelopsin	Catechin	Epicatechin	Triandrin	Caffeic acid der. I	Caffeic acid der. II	Purpurein I	Purpurein II	Salireposide	Syrengin		
*S. humboldtiana* (HU1)	0.69					0.01	0.33	0.40			0.09		1.47	1.24		2.98					0.26	0.65	8.12	2
*S. purpurea* (PU1)	1.27	11.41			17.25	0.10	3.01	1.61	0.29	0.10	0.06	1.10		3.39					0.05	0.11	1.14	0.36	41.25	4
**Crosses**																								
1	0.32	0.17			0.87	0.03	1.05	1.44			0.03	0.57	0.06	2.71		0.23					0.76	0.25	8.49	4
2	2.31	1.19				0.01	0.26	0.43			0.09			1.83								0.18	6.30	3
3	1.23	1.42				0.03	1.00	1.38	0.18	0.04	0.09	0.49		2.43		0.76					0.82	0.24	10.11	4
*S. alba* (AL2)	0.41		0.97								0.19	0.47		0.73	0.41	0.46	0.30					0.45	4.39	3
*S. x rubens* (AL1_h)			2.80		1.66					0.10	0.30			1.04	0.40	0.52	0.23					0.34	7.39	3
**Crosses**																								
1	0.96									0.10	0.17			1.29	0.45	0.09	0.68					0.25	3.99	3
2	0.55		1.55							0.05	0.12			1.06		0.12	0.56					0.21	4.22	3
*S. alba* (AL2)	0.41		0.97								0.19	0.47		0.73	0.41	0.46	0.30					0.45	4.39	3
*S. pentandra* (PE1)	0.16		33.22	4.38							0.03			0.41	0.25							0.34	38.79	3
**Crosses**																								
1	0.99		26.09	5.58							0.16		0.24	0.48			0.25					0.68	34.47	3
2	1.15		18.08	1.21							0.24	0.63	0.74	0.51			0.25					0.62	23.43	3
*S. pentandra* (PE2)	0.27		14.92	1.35							0.33			1.39		0.23	0.14					0.13	18.76	3
*S. lasiandra* (LA1)	0.72		2.11								0.08	0.41		1.32		0.60	0.25					0.65	6.13	3
**Crosses**																								
1	0.62		13.92								0.43			1.41		0.17	0.55					0.40	17.50	3
2	1.27										0.49			1.18			0.42					0.22	3.58	3
3			3.57								0.23			0.87		0.06	0.27					0.27	5.27	3
*S. pentandra* (PE2)	0.27		14.92	1.35							0.33			1.39		0.23	0.14					0.13	18.76	3
*S. alba* (AL5)	0.26										0.07			0.92		0.72	0.35					0.27	2.59	3
**Crosses**																								
1	1.01		10.03								0.19			1.91		1.25	0.51					0.21	15.11	3
2	2.93		4.15		3.07						0.35			2.86		1.72	0.30					0.26	15.64	3
3	0.93		19.45		2.87						0.10			1.42		0.06	0.26					0.19	25.28	3
4	0.22		9.92								0.53			1.50		0.42	0.36					0.31	13.26	3
*S. alba* (AL3)	1.12		1.12								0.11			1.49		0.34	0.56					0.38	5.12	3
*S. alba* (AL4)											0.13			0.91	0.57	0.39	0.32					0.52	2.84	3
**Crosses**																								
1	1.35		1.16								0.08			1.48	0.58		0.68					0.35	5.68	3
2	1.39										0.18			1.69	0.61		0.57					0.21	4.65	3

**glc, glucoside; der., derivate; S., Salix. Standard deviation of all compound contents varied between 0.00 and 0.16.**

**TABLE 4:** Phenolic compound profile of clones of *S. daphnoides*, *S. viminalis*, *S. purpurea, S. humboldtiana × S. purpurea*, and their crosses.

	Phenolic compounds (mg/g DW)																						

	Salicylates				Flavonoids								Flavan-3-ols		Other phenolic compounds							Total	Cluster targeted
				
	Salicin	Salicortin	2′-O-Acetylsalicortin	Tremulacin	Eriodictyol-7-glc	Naringenin-5-glc I	Naringenin-5-glc II	Naringenin-7-glc	Luteolin-7-glc	Quercetin-hexoside	Isosalipurposide	Ampelopsin	Catechin	Epicatechin	Triandrin	Caffeic acid der. I	Caffeic acid der. II	Purpurein I	Purpurein II	Salireposide	Syrengin		
*S. purpurea* (PU3)	2.39	2.61		1.62		2.42	3.64	0.07	0.09	0.14	1.66		1.47					0.12	0.15	3.50	0.98	20.86	4
*S. daphnoides* (DA6)	1.58	3.42		2.58	0.04	1.13	1.33	1.06	0.02		0.90		1.70	0.47		0.52	0.61				0.83	16.18	1
**Crosses**																							
1	0.93	2.17			0.06	1.28	1.62	0.46	0.16	0.23	0.72		1.64			0.10		0.05		0.48	0.99	10.89	4
2	2.04	5.21		4.54	0.03	1.09	1.38	0.55	0.20	0.15	0.87		2.87	0.42				0.02		0.57	1.40	21.35	4
3	1.52	4.13		4.70	0.02	0.87	1.06	0.33	0.11	0.17	0.68		1.60	0.26				0.02		1.15	0.66	17.28	4
4	1.25	4.18		6.30	0.08	1.05	1.58	0.40	0.07	0.12	0.80		2.73	0.32		0.09		0.01		1.36	0.84	21.18	4
5	0.98	3.32		1.45	0.02	0.95	1.32	0.37	0.03	0.07	0.75		2.15	0.28		0.12		0.05		0.56	0.49	12.91	4
*S. purpurea* (PU3)	2.39	2.61		1.62		2.42	3.64	0.07	0.09	0.14	1.66		1.47					0.12	0.15	3.50	0.98	20.86	4
*S. purpurea* (PU2)	1.01	3.54		8.09	0.45	2.44	2.12	0.46	0.28	0.22	1.37	0.11	3.58						0.07	2.12	0.44	26.30	4
**Crosses**																							
1	0.69	3.99		10.30	0.14	1.37	1.91	0.18	0.17	0.17	1.09	0.10	2.45							2.58	0.70	25.84	4
2	0.92	2.95		6.76	0.25	2.13	2.77	0.38	0.19	0.19	1.66	0.20	2.53						0.05	1.74	0.50	23.22	4
3	0.92	3.36		0.90	0.16	2.30	3.25	0.23	0.13	0.21	1.18		2.25					0.11	0.18	1.93	0.46	17.57	4
*S. humboldtiana* × *S. purpurea* (HU1 × PU1)	0.82				0.02	1.40	1.97		0.07	0.05			3.06		0.33			0.07	0.28	1.27	0.47	9.81	4
*S. daphnoides* (DA4)	1.45	4.98		3.45	0.10	1.36	1.66	2.07	0.04	0.03	2.95		1.62	0.95		1.14	0.99				1.71	24.50	1
**Crosses**																							
1	0.43	1.85		0.52	0.15	1.54	2.21	0.64	0.06	0.02	2.06		1.64		0.24	0.20	0.05				0.48	12.09	4
2	0.50	1.76			0.10	0.89	1.22	0.31	0.07	0.03	0.95		1.63		0.15	0.17	0.04				0.51	8.33	4
3	1.20	3.19		1.49	0.06	1.53	2.17	0.34	0.08	0.07	0.93		1.83		0.23	0.12					0.78	14.02	4
*S. purpurea* (PU3)	2.39	2.61		1.62		2.42	3.64	0.07	0.09	0.14	1.66		1.47					0.12	0.15	3.50	0.98	20.86	4
*S. viminalis* (*schwerinii × viminalis*) (VI3_h*)												0.46	0.78		10.37						0.36	11.97	2
**Crosses**																							
1	0.97	0.18	3.12	1.60	0.02	0.60	0.83		0.03	0.11	0.40	0.21	1.60		3.84		0.13	0.11	0.08	0.90	0.63	15.37	4
2		0.67	5.54	5.01	0.01	0.59	0.73			0.18	0.47	0.29	1.56		2.44			0.14	0.23	1.46	0.43	19.75	4

**glc, glucoside; der., derivate; S., Salix. Standard deviation of all compound contents varied between 0.00 and 0.16.**

Based on the clustering analysis (grouping of phenolic compounds found by targeted analysis) all investigated *Salix* clones (parent species and crosses) can be divided in four general compound clusters: cluster 1 (*S. daphnoides* including selected crosses of *S. daphnoides* with *S. viminalis* and *S. purpurea*), cluster 2 (*S. viminalis*, *S. humboldtiana*, *S. nigra* including selected crosses of *S. viminalis* with *S. daphnoides* and *S. nigra* with *S. pentandra*), cluster 3 (*S. pentandra*, *S. alba*, *S. x rubens*, *S. lasiandra* and crosses of these species, as well as HU1 × PU1_2, VI1 × DA1_9, VI6, and PU4 × VI2 crosses), cluster 4 (*S. purpurea* clones including selected crosses of *S. purpurea* with *S. daphnoides* or VI3_h, HU1 × PU1 and crosses with *S. daphnoides*, and HU1 × PU1_1,3). Clones belonging to cluster 1 generally had comparatively high contents of salicylates and other phenolic compounds such caffeic acid derivatives and syrengin, naringenin-5-*O*-glucoside as well as high contents of catechins (Table 1). Clones of cluster 2 were characterized by very high contents of the phenolic compound triandrin and high contents of the flavonoid ampelopsin (Table 2). Clones of cluster 3 were characterized by very high contents of the salicylates 2′-*O*-acetylsalicortin and 2′-*O*-acetylsalicin, but also flavonoids, catechins, and other phenolic compounds such as triandrin and salireposide (Table 3). Clones of cluster 4 showed high contents of the salicylates salicin, salicortin, and tremulacin as well as catechins and had also very high contents of flavonoids such as naringenin-7-*O*-glucoside and isosalipurposide (Table 4).

## Discussion

Based on the targeted metabolomics approach, inter- and intraspecific crosses of *Salix* species showed mostly an intermediate total secondary plant metabolite content of their parents (Tables 1–4). Focusing on single compounds, *S. daphnoides* has high levels of salicortin and tremulacin, which is missing in *S. viminalis*. The obtained crosses contained these compounds (for example VI1 × DA1, Table 2). According to this, the crosses clustered similar to their parental forms [exceptions: VI1 × DA1_9, PU4 × VI2_1-2, HU1 × PU1_2, PU3 × DA6_1-5 (HU1 × PU1) × DA4_1-3]. This is in accordance to former analysis of Orians et al. (2000) who detected that two phenolic glycosides in the leaves of hybrids of *Salix eriocephala* and *Salix sericea*, salicortin and 2′-cinnamoylsalicortin, had intermediate contents. However, the authors found high variations in compound contents for hybrids but on average lower than the midpoint of the parental taxa. Orians et al. (2000) concluded an incomplete dominance in inheritance of phenolic glycosides and therefore, more included alleles that control the production of these compounds. Moreover, Hallgren et al. (2003) found intermediate contents of condensed tannins and phenolic glucosides in hybrids of *Salix caprea* and *Salix repens* in comparison to the parental generation. In some cases, crosses show lower compound contents, for example identified for quercetin-hexoside in DA2 × DA3 (Table 1) or for salicin in most crosses of VI1 × DA1 (Table 2). Higher compound contents could be observed for example for salicortin or tremulacin in (DA2 × DA3) × VI2, Table 1. These results could be traced back to a heterosis effects (over-dominance) as identified for condensed tannins in eucalypt hybrids (O’Reilly-Wapstra et al., 2014). Nevertheless, tending reasons could be differences in plant age and therefore a different shoot amount and rootstock development, but also diverse environmental conditions on the growth locations or variable season of harvest of *Salix* species (Förster et al., 2008). Other inheritance pattern like dominance or co-dominance (similar contents to one or both parental taxa) and under-expression (lower contents in hybrids) are also common for secondary plant metabolites (Cheng et al., 2011). For instance, this can be observed for VI1 × DA1 (Table 2) where the metabolite profile of cross 1 is comparable to the profile of DA1, whereas the crosses 2–8 were similar to VI1. The results obtained are in accordance with Rieseberg and Ellstrand (1993), who showed that 68% of the chemicals produced by the parents can be found in hybrids. Additionally, hybrids can lack in specific secondary plant metabolites (deficiency) or can show novel compounds which cannot be identified in the parental generation (Orians, 2000). Focusing on the quality and not on the quantity of plant compounds, according to the author, 5% of the chemicals identified in the parental generation are new in hybrids and 27% of chemicals get lost. These findings are in accordance with our results showing that 22% of the analyzed phenolics which were identified in at least one of the parents could not be detected in the *Salix* crosses. If both parents show the metabolite, just 2% get lost in the filial generation. In case of the identified compounds in the present study, we found, for example, that the analyzed crosses of AL3 and AL4 do not show triandrin, whereas in both parents triandrin could be identified (Table 3). In crosses of AL2 and PE1, ampelopsin was detected as a novel compound, which was not detected in the parental generation. New compounds in hybrids, which were not detected in the parental generation, could be hardly recognized in *Salix*. Therefore, we can assume that most chemical compounds were inherited dominantly with Mendelian laws. However, it has been postulated that male *Salix* plants were affected less severe by fungal pathogens (*Melampsora* spp.) in comparison to females (Moritz et al., 2016). Apparently, plant characteristics can be inherited gender-dependent. A different vulnerability of female and male *Salix* clones to fungal diseases could be explained by a different secondary plant metabolite profile even if other authors could not identify such a relationship (Hakulinen, 1998; Hakulinen and Julkunen-Tiitto, 2000). In our study, the analyzed crosses of the filial generation did not differ clearly between male and female descendants in their secondary metabolite pattern. Additionally, it was not observed that one parent has generally more influence on the metabolite pattern of the *Salix* crosses in the filial generation.

Important to keep in mind is that the profile of secondary plant metabolites is not only genetically specific, it can also be influenced by biotic as well as abiotic factors like pest infestations and environmental conditions (e.g., temperature, soil). It is therefore not surprising that selected *Salix* clones should be cultivated under controlled conditions to form a stable and safe source of bark with high quality for medical uses (Sulima and Przyborowski, 2019). In our study, it was not possible to grow all 92 *Salix* genotypes in one site. Therefore, clones were not cultivated under controlled conditions, but under similar environmental conditions in Berlin/Brandenburg, Germany. Sulima et al. (2017) analyzed bark extracts from 91 genotypes of *S. purpurea* and identified a high variation in the content of salicylates between genotypes. In own investigations a high intraspecific variability of bark phenolics of *Salix* cultivated in the same site was identified, too (Förster, 2010). Therefore, it seems to be advisable to control the cultivation conditions strongly. Due to the amount of 92 different clones (and therefore approximately 800 plants) included in this study, it was not possible to grow all *Salix* clones at one side. To minimize the influence of this factor for the present study, nearly all clones analyzed were cultivated at two sites, in Berlin or Zepernick. Additionally, for the standardization of willow bark extracts, which is absolutely necessary for the production of efficient bark extract, specific components should be analyzed to evaluate therapeutic properties (Sulima and Przyborowski, 2019).

Crosses were generated to obtain descendants, which perform better than their parents (heterosis effect). For *Salix*, targeted crossing (interspecific hybridization) is used to generate genotypes with a higher yield and biomass production due to their use as energy crop as well as plants with an increased vigor, pest and disease resistance. Hybridization plays therefore a key role to obtain improved genotypes which can be used as bioenergy crop (Carlson, 2018). Additionally, generating intra- and interspecific crosses of *Salix* offer the chance to generate genotypes with new combinations of secondary plant metabolites in the bark. Analysis of heterosis for vitamins and antioxidant pigments in cauliflower showed a positive direction for ascorbic acid, anthocyanin, and lycopene concentration and a negative direction for carotenoids and chlorophyll pigments (Dey et al., 2014). Therefore, hybrids show different contents of vitamins and antioxidant plant pigments. Analysis with grape hybrids showed a substantial positive mid-parent and better-parent heterosis for anthocyanins and total flavonoids, whereas a negative mid-parent heterosis and heterobeltiosis was found for total phenolics (Sahoo et al., 2017). Based on these findings from literature as well as our results of the analysis of targeted compounds in the *Salix* bark, it is possible to generate *Salix* crosses with a different profile of distinct secondary plant compounds. Due to diverse health promoting effects of the compounds as well as possible synergetic effects, a different bioactivity of generated bark extracts is obvious. To define possible pharmaceutical effects, it is needed to evaluate the quantity of phenolic acids, flavan-3-ols, flavonoids, salicylates, and other phenolic compounds specific for *Salix* species and link it to bioactivity in bioassays, which is currently ongoing in our collaborative project.

Based on chemoprofiling of the *Salix* clones of parent species and crosses performed by use of the targeted approach, genotypes could be grouped into different clusters with similar compound profiles. Mostly, crosses show a similar phenolic compound profile as their parents (same cluster as both or one parent; two exceptions: PU4 × VI2_1-2, Table 2; HU1 × PU1_2, Table 3). However, targeted crossing resulted in hybrids from species with different chemical profiles (descendants in different cluster, VI1 × DA1, Table 2; HU1 × PU1, Table 3). Based on the Mendelian laws mixed compound profiles have been obtained. In further breeding approaches, the focus should be set on the enhancement of single compounds with most health promoting effects or specific compound combinations. Due to the fact that there are other potent bioactive compounds in the *Salix* bark (f. e. Nahrstedt et al., 2007) except salicylates, the full therapeutic potential of Salicis cortex can be reached through a targeted selection of clones used for extract production. The compound profile in the bark is strongly species dependent and can vary in inter- and intraspecific crosses qualitatively and quantitatively. Generating different chemoprofiles, and therefore new combinations of secondary plant metabolites, offer the chance to identify *Salix* crosses with improved effects on human health.

The presented study included the qualitative and quantitative analysis of possible literature-known bioactive compounds (*Salix* characteristic and known for their health promoting potential) in a targeted metabolomics approach and is a very useful starting point for crossing experiments. The results form one important factor for clustering different chemotypes of *Salix*. As other bioactive substances may be present in willow bark and could be a second important factor for breeding strategies, an untargeted approach was performed (Supplementary Information). In sum, five groups (four main groups and one group just consisting of one clone) could be visualized via PCA analysis of the untargeted metabolomics data (Supplementary Figure). Cluster and groups identified by the targeted (Tables 1–4) and untargeted approach (Supplementary Figure) have been very similar with many overlaps. Those cluster/groups are very good starting points for genotype selection to perform bioactivity assays. Bark extracts of selected clones from the four cluster/groups as well as fractions thereof were analyzed in bioactivity assays; results will be published elsewhere. To identify further unknown potential bioactives in the *Salix* bark, an activity guided fractionation is currently in progress. The combination of an untargeted and a targeted metabolomics approach presents a valuable opportunity for a rapid fingerprint of different plants, extracts, or species as postulated for *Rosmarinus officinalis* by Maldini et al. (2016).

## Data Availability Statement

The raw data supporting the conclusions of this article will be made available by the authors, without undue reservation, to any qualified researcher.

## Author Contributions

NF, KA, MZ, CD, and IM conceived and designed the experimental trial. MZ conducted the plant breeding. NF harvested and prepared plant material. NF, KA, and SB did analytical lab work. NF, KA, VM-K, and SB analyzed the data. NF wrote the manuscript with the assistance of KA, VM-K, CD, and IM. All authors read and approved the manuscript.

## Conflict of Interest

The authors declare that the research was conducted in the absence of any commercial or financial relationships that could be construed as a potential conflict of interest.
