# Supplementary material for: Chemoprofiling as Breeding Tool for Pharmaceutical Use of Salix
Source: Front Plant Sci. 2021 Apr 1;12:579820. doi: 10.3389/fpls.2021.579820 (PMC8047421; doi:10.3389/fpls.2021.579820)
Supplement: Supplementary file 1 [file Data_Sheet_1.pdf]

SUPPORTING INFORMATION

Chemoprofiling as breeding tool for pharmaceutical use of *Salix*

Nadja Förster[1*], Kyriaki Antoniadou[2], Matthias Zander[1], Sebastian Baur[2], Verena Karolin Mittermeier-Kleßinger[2], Corinna Dawid[2,3], Christian Ulrichs[1], Inga Mewis[1]

[1]Division Urban Plant Ecophysiology, Humboldt-Universität zu Berlin, Berlin, Germany
[2]Chair of Food Chemistry and Molecular Sensory Science, Technical University of Munich, Freising, Germany
[3]Bavarian Center for Biomolecular Mass Spectrometry, Technical University of Munich, Freising, Germany

Supp. Table: Origin of *Salix* species and crosses

Species	Abbreviation	Cultivation	Origin
S. viminalis	VI1	ZP	chance seedling Waldsieversdorf 2011, G
S. daphnoides	DA1	DA	G, Mecklenburg-Vorpommern, Pampow
S. viminalis x *S. daphnoides*	VI1xDA1_1	DA	new cross HU Berlin 2014, G
S. viminalis x *S. daphnoides*	VI1xDA1_2	DA	new cross HU Berlin 2014, G
S. viminalis x *S. daphnoides*	VI1xDA1_3	DA	new cross HU Berlin 2014, G
S. viminalis x S. daphnoides	VI1xDA1_4	DA	new cross HU Berlin 2014, G
S. viminalis x *S. daphnoides*	VI1xDA1_5	DA	new cross HU Berlin 2014, G
S. viminalis x *S. daphnoides*	VI1xDA1_6	DA	new cross HU Berlin 2014, G
S. viminalis x *S. daphnoides*	VI1xDA1_7	DA	new cross HU Berlin 2014, G
S. viminalis x *S. daphnoides*	VI1xDA1_8	DA	new cross HU Berlin 2014, G
S. viminalis x *S. daphnoides*	VI1xDA1_9	DA	new cross HU Berlin 2014, G
S. viminalis	VI2	Wriezen	Swedish clone, 'Jorr', sold by Lantmännen Agroenergie AB
S. daphnoides	DA2xDA3	ZP	new cross HU Berlin 2011, G
S. daphnoides x *S. viminalis*	(DA2xDA3)xVI2_1	DA	new cross HU Berlin 2014, G
S. daphnoides x *S. viminalis*	(DA2xDA3)xVI2_2	DA	new cross HU Berlin 2014, G
S. daphnoides **x** *S. viminalis*	(DA2xDA3)xVI2_3	DA	new cross HU Berlin 2014, G
S. daphnoides x *S. viminalis*	(DA2xDA3)xVI2_4	DA	new cross HU Berlin 2014, G
S. daphnoides x *S. viminalis*	(DA2xDA3)xVI2_5	DA	new cross HU Berlin 2014, G
S. humboldtiana x *S. purpurea*	HU1xPU1	ZP	new cross Waldsieversdorf 2011, G
S. daphnoides	DA4	DA	G, Mecklenburg-Vorpommern, Zarrendorf
(S. humboldtiana x *S. purpurea)* x *S. daphnoides*	(HU1xPU1)xDA4_1	DA	new cross HU Berlin 2014, G
(S. humboldtiana x *S. purpurea)* x *S. daphnoides*	(HU1xPU1)xDA4_2	DA	new cross HU Berlin 2014, G
(S. humboldtiana x *S. purpurea)* x *S. daphnoides*	(HU1xPU1)xDA4_3	DA	new cross HU Berlin 2014, G
S. viminalis (schwerinii x *viminalis)*	VI3_h	WS	Swedish clone 'Olof', sold by Lantmännen Agroenergie AB
S. viminalis	VI4	DA	Swedish clone '79036' breeding company Svalöf-Weibull AB
S. viminalis x *S. viminalis (schwerinii* x *viminalis)*	VI4xVI3_1	ZP	new cross Waldsieversdorf 2012, G
S. viminalis x *S. viminalis (schwerinii* x *viminalis)*	VI4xVI3_2	ZP	new cross Waldsieversdorf 2012, G
S. alba x *S. fragilis* (*S.* x *rubens*)	AL1_h	ZP	Hungary, Baja, 'B38', University of Sopron
S. alba	AL2	ZP	G, Thüringen, Erfurt, Höngeda
S. alba x *S. alba* x *S.* x *rubens*	AL2xAL1_1	DA	new cross Waldsieversdorf 2014, G
S. alba x *S. alba* x *S.* x *rubens*	AL2xAL1_2	DA	new cross Waldsieversdorf 2014, G
S. pentandra	PE1	DA	G, Brandenburg, Eggersdorf
S. alba x *S. pentandra*	AL2xPE1_1	DA	new cross Waldsieversdorf 2014, G
S. alba x *S. pentandra*	AL2xPE1_2	DA	new cross Waldsieversdorf 2014, G
S. alba	AL3	ZP	G, Brandenburg, Waldsieversdorf
S. alba	AL4	ZP	Romania, Bukarest, Institutul de Cercetări Forestiere
S. alba x *S. alba*	AL3xAL4_1	DA	new cross Waldsieversdorf 2014, G
S. alba x *S. alba*	AL3xAL4_2	DA	new cross Waldsieversdorf 2014, G
S. viminalis	VI5	DA	English clone, 'Bowles', UK National Willows Collection
S. viminalis x *S. viminalis*	VI5xVI2_1	DA	new cross Waldsieversdorf 2011, G
S. viminalis x *S. viminalis*	VI5xVI2_2	DA	new cross Waldsieversdorf 2011, G
S. viminalis x *S. viminalis*	VI5xVI2_3	DA	new cross Waldsieversdorf 2011, G
S. viminalis x *S. viminalis*	VI5xVI2_4	DA	new cross Waldsieversdorf 2011, G
S. daphnoides	DA3	DA	G, Baden-Württemberg, Laimnau Argen
S. daphnoides	DA2	DA	Poland, Westpommern, Miedzyzdroje

S. daphnoides	DA2xDA3_1	DA	new cross HU Berlin 2011, G
S. daphnoides	DA2xDA3_2	DA	new cross HU Berlin 2011, G
S. daphnoides	DA2xDA3_3	DA	new cross HU Berlin 2011, G
S. daphnoides	DA2xDA3_4	DA	new cross HU Berlin 2011, G
S. daphnoides	DA2xDA3_5	DA	new cross HU Berlin 2011, G
S. daphnoides	DA2xDA3_6	DA	new cross HU Berlin 2011, G
S. daphnoides	DA2xDA3_7	DA	new cross HU Berlin 2011, G
S. daphnoides	DA2xDA3_8	DA	new cross HU Berlin 2011, G
S. daphnoides	DA5	DA	Poland, Westpommern, Dziwnow
S. purpurea	PU2	DA	G, Baden-Württemberg, Birkenried Pfohren
S. daphnoides x *S. purpurea*	DA5xPU2_1	ZP	new cross HU Berlin 2012, G
S. daphnoides x *S. purpurea*	DA5xPU2_2	ZP	new cross HU Berlin 2012, G
S. lasiandra	LA1	ZP	USA, Maryland, National Plant Materials Center Beltsville, Soil Conservation Service
S. pentandra	PE2	DA	Austria, Salzburg, Zell am See, Zeller Moos
S. pentandra x *S. lasiandra*	PE2xLA1_1	DA	new cross HU Berlin 2014, G
S. pentandra x *S. lasiandra*	PE2xLA1_2	DA	new cross HU Berlin 2014, G
S. pentandra x *S. lasiandra*	PE2xLA1_3	DA	new cross HU Berlin 2014, G
S. alba	AL5	ZP	G, Mecklenburg-Vorpommern, Schloen
S. pentandra x *S. alba*	PE2xAL5_1	DA	new cross HU Berlin 2013, G
S. pentandra x *S. alba*	PE2xAL5_2	DA	new cross HU Berlin 2013, G
S. pentandra x *S. alba*	PE2xAL5_3	DA	new cross HU Berlin 2013, G
S. pentandra x *S. alba*	PE2xAL5_4	DA	new cross HU Berlin 2013, G
S. daphnoides	DA6	DA	G, Mecklenburg-Vorpommern, Zarrendorf
S. purpurea	PU3	ZP	G, Bayern, Miesbach, Aschenbach
S. purpurea x *S. daphnoides*	PU3xDA6_1	DA	new cross Waldsieversdorf 2014, G
S. purpurea x *S. daphnoides*	PU3xDA6_2	DA	new cross Waldsieversdorf 2014, G
S. purpurea x *S. daphnoides*	PU3xDA6_3	DA	new cross Waldsieversdorf 2014, G
S. purpurea x *S. daphnoides*	PU3xDA6_4	DA	new cross Waldsieversdorf 2014, G
S. purpurea x *S. daphnoides*	PU3xDA6_5	DA	new cross Waldsieversdorf 2014, G
S. purpurea x *S. viminalis (schwerinii* x *viminalis)*	PU3xVI3_1	ZP	new cross Waldsieversdorf 2012, G
S. purpurea x *S. viminalis (schwerinii* x *viminalis)*	PU3xVI3_2	ZP	new cross Waldsieversdorf 2012, G
S. purpurea x *S. purpurea*	PU3xPU2_1	ZP	new cross Waldsieversdorf 2012, G
S. purpurea x *S. purpurea*	PU3xPU2_2	ZP	new cross Waldsieversdorf 2012, G
S. purpurea x *S. purpurea*	PU3xPU2_3	ZP	new cross Waldsieversdorf 2012, G
S. purpurea	PU4	DA	G, Bayern, Weilheim-Schongau, Ammer
S. purpurea x *S. viminalis*	PU4xVI2_1	DA	new cross Waldsieversdorf 2011, G
S. purpurea x *S. viminalis*	PU4xVI2_2	DA	new cross Waldsieversdorf 2011, G
S. humboldtiana	HU1	WS	Swedish clone, 'SH2' breeding company Svalöf-Weibull AB
S. viminalis	VI6	WS	Swedish clone, '78195' breeding company Svalöf-Weibull AB
S. humboldtiana x *S. viminalis*	HU1xVI6_1	ZP	new cross Waldsieversdorf 2012, G
S. humboldtiana x *S. viminalis*	HU1xVI6_2	ZP	new cross Waldsieversdorf 2012, G
S. purpurea	PU1	DA	G, Bayern, Garmisch-Partenkirchen, Oberau
S. humboldtiana x *S. purpurea*	HU1xPU1_1	DA	new cross Waldsieversdorf 2011, G
S. humboldtiana x *S. purpurea*	HU1xPU1_2	DA	new cross Waldsieversdorf 2011, G
S. humboldtiana x *S. purpurea*	HU1xPU1_3	DA	new cross Waldsieversdorf 2011, G
S. nigra	SN1	Garzau	not known
S. nigra x *S.pentandra*	SN1xPE1	ZP	new cross Waldsieversdorf 2012, G

G = Germany; DA = Berlin-Dahlem, Germany; ZP = Zepernick, Germany, h = hybrid

Instrumental parameters and workflow of untargeted analysis:

Chromatographic conditions

Extracts used for the untargeted analysis were produced by extracting 10 mg freeze-dried, powdered willow bark with 500 µL 70% methanol (0.1% formic acid) in an ultrasonic bath with ice water for 15 min. After centrifugation (10.000 rpm, 5 min, 20 °C), the supernatant was collected and the pellet was re-extracted with 200 µL of the extraction solution twice. The combined supernatants were filled with ultrapure water up to 1 ml and filtered using SpinX tubes (0.22 µm). For chemoprofiling of the selected 92 *Salix* bark extracts, UHPLC-ESI$^-$-IMS-TOF-MS (Waters, Manchester, UK) data were used to carry out a principal component analysis (PCA). The used Acquity i-class UHPLC system (Waters, Milford, MA, USA) consisted of a sample manager, column oven, and a binary solvent manager (Waters, Milford, MA, USA) coupled with a Vion IMS QTOF mass spectrometer (Waters, Manchester, UK) and operated with the UNIFI v1.8 software (Waters, Milford, MA, USA). Separation was performed on an Acquity UHPLC BEH C18 column (1.7 µm, 130 Å, 2.1 mm x 50 mm, 3/pkg; Waters, Manchester, UK) at a column temperature of 45 °C. The eluents of the mobile phase were A) 0.1% formic acid in ultrapure water and B) 0.1% aqueous formic acid in acetonitrile. The injection volume was 1 µL and the following gradient program was used: 1% B (0 - 1.0 min), 1 - 60% B (1 - 4.5 min), 60 - 80% B (4.5 - 5.5 min), 80 - 100% B (5.5 - 6.0 min), 100% B (6.0 - 7.0 min), 100 - 1% B (7.0 - 7.5 min), 1% B (7.5 - 8.0 min). The analysis of each of the 92 bark extracts (MeOH/0.1% formic acid (70/30, v/v)) was performed with a flow rate of 0.4 mL/min injecting each sample four times (technical replicates). For quality control (QC reference), a pooled sample of all 92 bark extracts, and a blank was injected 20 times and 10 times, respectively, throughout the run time to ensure a consistent analysis. Moreover, the quality control was used for the automatic normalization in the Progenesis QI v2.1 software (Waters, Manchester, UK) and thus error correction of the detected MS signals.

MS conditions

The ion source was operated in the HDMS^e sensitivity mode via negative electrospray ionization (ESI). The scan time for the HDMS^e experiment was set to 0.3 s. Following ion source parameters were applied: capillary voltage 2.50 kV, source temperature 150 °C, desolvation temperature 450 °C, cone gas flow 50 L/h, desolvation gas flow 900 L/h.
The collision energy was ramped from 20 to 40 eV. The QTOF system was calibrated from *m/z* 50 - 1000 using a MajorMix (Waters, Milford, MA, USA) solution. To provide tuning and calibration of the MS system a 50 pg/100 µL solution of pentapeptide leucine enkephalin (Tyr-Gly-Gly-Phe-Leu, *m/z* 554.2615 [M-H]$^-$; Merck KGaA, Darmstadt, Germany) in ACN/0.1% formic acid (1/1, v/v) was used for lock mass correction. This reference solution was infused every 0.5 min with a scan of 2 s.

Mass Data Processing and Analysis

The Progenesis QI v2.1 software was applied for data processing. In total, 396 profile MS^e raw data were imported and processed automatically. Subsequently, chromatographic peak alignment, experimental design setup, peak picking, deconvolution, compound identification, and compound statistics were executed. Peak picking was employed by following parameters: all runs, automatic limits, default sensitivity, retention time limits (from 0.05 to 7.5 min), and fragment sensitivity (base peak 1%). Of all the analyzed QC references, Progenesis software picked automatically the quality control reference 7 as the most suitable QC sample. This allowed normalization and comparison between groups or *Salix* crosses. Subsequent adduct forms were selected: [M-2H]$^-$, [M-H_2O-H]$^-$, [M-H]$^-$, [M+FA-H]$^-$, [2M-H]$^-$, [2M+FA-H]$^-$, [2M+HAc-H]$^-$, and [3M-H]$^-$. Tag filtration was carried out by means of ANOVA p-value ≤ 0.05 and Max-fold change ≥ 2 to identify

significant compound differences between the groups. To compare the fragmentation patterns of the analysis, an *in silico* fragment database was created by defining the MetaScope search parameters using an automatic detection format: in-house compound database, auto-detect data format, precursor tolerance of 5 ppm, and theoretical fragmentation with fragment tolerance of 5 ppm as a fragment search method. The statistical analysis including the PCA was employed by means of EZinfo v3.0 (Umetrics, Sweden) conjugated with Progenesis QI using the Pareto scaling model.

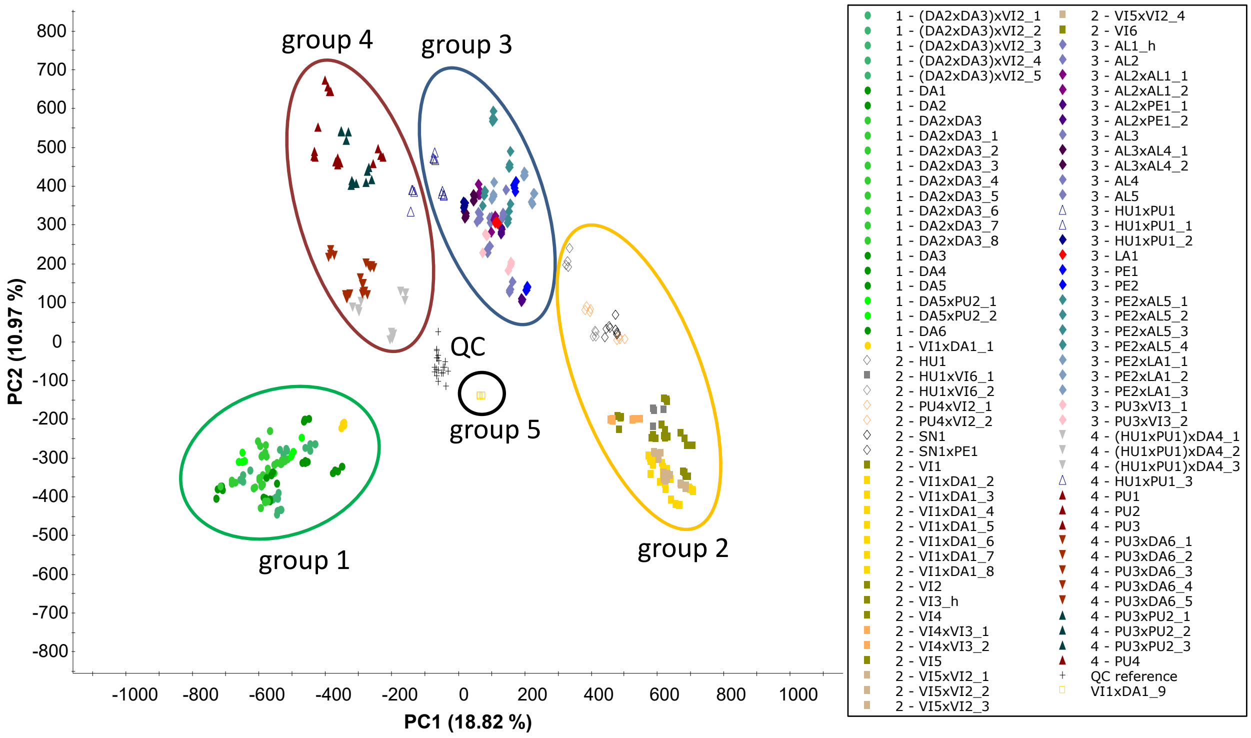

Supp. Figure: PCA score plot of 92 *Salix* genotypes. According to PCA analysis of untargeted UHPLC-TOF-MS data the different genotypes can be divided into five groups. Different colours and shapes refer to different *Salix* crosses as well as QC reference.
